# Synergistic effects of beneficial microbial inoculants and SMS-amendments on improving soil properties and Pinus seedling growth in degraded soils

**DOI:** 10.3389/fmicb.2025.1608689

**Published:** 2025-09-01

**Authors:** Xuan Bo, Yong Liu, Hong Zhang, Chao Su, Yang Miao

**Affiliations:** ^1^Institute of Loess Plateau, Shanxi University, Taiyuan, China; ^2^College of Environmental and Resource Sciences, Shanxi University, Taiyuan, China; ^3^College of Materials Science and Engineering, Taiyuan University of Technology, Taiyuan, China

**Keywords:** degraded soil, soil remediation, waste composting, mixed microbial inoculant, physicochemical properties, microbial diversity

## Abstract

Spent mushroom substrate (SMS) is a promising organic amendment for remediating degraded soils, yet its effectiveness is often limited by poor compost maturity and stability. This study aimed to enhance the quality and functionality of SMS compost through microbial inoculation and to evaluate its impact on soil improvement and plant growth. Three plant growth-promoting strains [*Bacillus subtilis (B. subtilis)*, *Azotobacter chroococcum (A. chroococcum)*, and *Paenibacillus mucilaginosus (P. mucilaginosus)*] and their combination as a mixed microbial inoculant (MMI) were used in composting. Subsequent pot experiments assessed changes in soil physicochemical properties, nutrient levels, microbial diversity, and the growth of *Pinus sylvestris* seedlings. Results showed that SMS amended with MMI significantly improved soil porosity, pH, and nutrient content, while enriching beneficial microbial communities dominated by *Proteobacteria* and *Basidiomycota*. Moreover, MMI treatment notably enhanced plant height, stem diameter, and chlorophyll content compared to control treatments. These findings highlight the synergistic effect of mixed microbial inoculants in optimizing SMS compost and promoting soil and plant health. The approach offers a sustainable strategy for the circular reuse of agricultural waste and effective restoration of degraded soils.

## 1 Introduction

Soil degradation is a serious threat to global food security, ecological balance, and climate resilience (Efthimiou, 2025). High productivity depends on effective soil management, which is closely linked to soil microbial activity. Soil microorganisms play a crucial role in nutrient cycling, organic matter decomposition, and maintaining soil health (Hu et al., 2024; Qiao et al., 2024). Unsustainable farming practices harm soil microbes, leading to degraded soil structure (Rombel et al., 2022), heavy metal accumulation (Taghavi et al., 2024), and biodiversity loss (Zhu et al., 2024). These effects significantly reduce the ecological services provided by soils. In China, this issue is particularly urgent due to the combined pressures of intensive cultivation and limited arable land. The degraded soil used in this study—classified as yellow-brown soil—has low organic matter, poor structure, weak water retention, and low microbial diversity. These issues limit its fertility and make recovery difficult using conventional methods.

In response to these challenges, the Chinese government advocates reducing synthetic fertilizers and pesticides while encouraging the use of organic fertilizers and biopesticides (Wang et al., 2018). These policies show the urgent need for sustainable soil rehabilitation.

As part of the circular economy, agricultural wastes such as SMS hold potential for soil improvement due to their resource recycling value. In China, approximately 15 million tons of SMS are produced annually (Carpio et al., 2023). However, untreated SMS contains molds and hazardous microbes, posing risks of soil and water contamination (Qiao et al., 2023). Incineration is commonly used but generates harmful pollutants such as dioxins (Kamal Baharin et al., 2022). Thus, sustainable and safe strategies for SMS reuse are urgently needed.

SMS has demonstrated strong potential in remediating degraded soils by reducing reliance on synthetic fertilizers, lowering heavy metal toxicity, and enhancing resistance to drought and diseases (Wang H.-W. et al., 2020). These effects are attributed to its favorable characteristics, including low bulk density, porous structure, and high organic nutrient content. High-temperature composting is typically applied to SMS to eliminate pathogens and accelerate organic matter degradation (Wang et al., 2017; Dabrowska et al., 2021). However, challenges such as unstable compost quality and insufficient maturity limit its application (Chen, 2022; Qiao et al., 2023). Improving compost stability and maturity is therefore critical to fully unlocking the potential of SMS as a soil amendment. Mature SMS has been shown to improve soil structure by increasing pH, promoting the formation of soil aggregates, and raising humus and nutrient content (Yu et al., 2018; Li X. et al., 2023). These improvements contribute to greater microbial diversity, better root development, and enhanced soil multifunctionality (Medina et al., 2012; Paula et al., 2017). Moreover, SMS slowly releases nutrients and mineralizes over time, making it ideal for long-term soil use (Lou et al., 2015). It may also reduce pesticide use by promoting beneficial microbes (Ribas et al., 2009).

To overcome the limitations of compost stability and maturity in SMS treatment, microbial inoculants such as *B. subtilis*, *A. chroococcum*, and *P. mucilaginosus* have been widely used. These functional microbes enhance compost quality by accelerating organic matter degradation, stabilizing nutrient transformation, and improving microbial community structure (Watabe et al., 2004; Tao et al., 2015; Li H. et al., 2023). Mixed microbial inoculants (MMIs) combine multiple beneficial strains and generally perform better than single strains by building more complex microbial communities (Xu et al., 2022). Despite promising composting performance, current research has largely focused on fermentation efficiency and nutrient conversion. The downstream impacts of MMI-enhanced SMS compost—particularly on soil physicochemical properties, microbial community dynamics, and plant development in degraded soils—remain insufficiently understood. This study aims to fill these knowledge gaps by assessing how SMS composts with different microbial inoculants affect soil restoration and plant growth.

In this study, SMS was combined with different microbial inoculants to investigate its effects on soil physicochemical properties, microbial diversity, and plant growth in degraded soil. The study aimed to achieve several objectives: (1) to develop an optimized SMS compost formulation with microbial inoculants; (2) to evaluate the influence of SMS amendments on the physicochemical properties of degraded soil; (3) to examine changes in microbial community composition and diversity; and (4) to assess the impact of SMS treatments on plant growth indicators and their potential for sustainable replanting This study not only aims to improve the composting process of SMS with microbial inoculants but also provides new perspectives on the sustainable utilization of mushroom waste for restoring degraded soils. The results are expected to contribute valuable insights for developing eco-friendly soil amendments and advancing sustainable agricultural practices.

## 2 Materials and methods

### 2.1 Site description and research stage

The experiment took place from 2021 to 2022 at the Guandi Mountain National Experimental Forestry Farm in Luliang City, Shanxi Province, China (37°23′–37°32′N, 111°24′–111°36′E). The area is located at an elevation of 1,300–2008 m and has a typical subtropical monsoon climate. Annual precipitation is less than 500 mm, with an average temperature of 4–6°C and a frost-free period of 120 days. The mean daily temperature exceeds 10°C, and the effective cumulative temperature ranges from 2,500–3,000°C. The soil is classified as yellow-brown soil. Detailed physicochemical properties and nutrient contents of the surface soil layer (0–20 cm) are presented in Supplementary Table S1.

SMS composting has two stages: the first stage uses aerobic composting with existing microorganisms at temperatures above 50°C, while the second stage adds microbial inoculants and keeps the temperature below 40°C.

Visual management of the composting process is achieved by measuring the changes in the key indicators of temperature during the composting process, so as to determine whether the composting is completed at each stage. When the temperature approaches ambient temperature, it indicates that the composting process is complete.

### 2.2 Microbial inoculant: properties and preparation

*Bacillus subtilis*, *A. chroococcum* and *P. mucilaginosus* were purchased from the China Industrial Microbial Strain Preservation and Management Center, and the chemical reagents used in the experiments were of analytically pure grades and were purchased from Aladdin Reagent Company.

The above strains belongs to Plant Growth-Promoting Microorganisms (PGPMs), which perform nitrogen fixation (*A. chroococcum*), phosphorus solubilization (*P. mucilaginosus*), and disease resistance (*B. subtilis*), respectively, and collectively promote plant growth. Its mechanism mainly involves the secretion of plant growth hormones (e.g., IAA) and antibiotic substances, which enhance the availability of soil nutrients like nitrogen and phosphorus while inhibiting pathogenic bacteria, thereby improving plant disease resistance and growth vigor.

Single colonies grown on agar medium were inoculated into the corresponding liquid medium, shaken in a constant temperature shaker at 180 rpm, and cultured at 30°C over a 24–72 h period (concentration of 1 × 10^8^–10^9^ colony-forming units (CFU)/mL) as a seed solution. The culture medium formula of different microbial inoculants is shown in Supplementary Table S2.

### 2.3 Composting procedure

The SMS (79% wood chips, 20% wheat bran, 1% gypsum) was purchased from a shiitake mushroom production workshop in Yangqu County, Shanxi Province, and dried until the moisture content was approximately 15%. Supplementary Table S3 displays the basic physicochemical properties of SMS.

SMS composting consists of two stages, and its quality can be comprehensively assessed using a combination of physical, chemical, and biological indicators. Mature compost is typically characterized by a dark brown color, loose texture, uniform particle size, low bulk density, and moderate moisture content. These attributes contribute to improved soil moisture retention and create favorable conditions for microbial activity. Chemically, mature compost generally exhibits a neutral to slightly acidic pH, high organic matter content, and significant concentrations of essential nutrients such as nitrogen, phosphorus, and potassium. These characteristics reflect its strong nutrient-supplying capacity, which is beneficial for plant growth. Biologically, high microbial activity, a diverse microbial community structure, and the presence of plant growth-promoting microorganisms are indicative of compost stability. These features also suggest good biosafety and considerable potential for application in soil improvement.

### 2.4 Experimental design and implementation

In this study, a systematic assessment was conducted to evaluate how different microbial inoculants influence the composting performance of spent mushroom substrate (SMS), using a two-stage experimental setup.

Six treatments were set up: (1) no SMS soil amendment (CK); (2) SMS soil amendment with no microbial inoculant (C1); (3) SMS soil amendment with *B. subtilis* (C2); (4) SMS soil amendment with *A. chroococcum* (C3); (5) SMS soil amendment with *P. mucilaginosus* (C4); and (6) SMS soil amendment with mixed microbial inoculant (V(*B. subtilis*): V(*A. chroococcum*): V(*P. mucilaginosus*) = 1:1:1) (C5). The study utilized a randomized block design, with three treatment replicates per block (Table 1). Treatment C1, which involved SMS compost without microbial inoculants, was designed to isolate the effects of microbial inoculation while accounting for the transformation and stabilization brought about by the composting process itself. A treatment using unprocessed, non-composted SMS was not included, as direct application of raw SMS may introduce unstable organic compounds, phytotoxicity, or adverse microbial interactions, which do not reflect common agricultural practices. Therefore, the comparison between C1 and C2–C5 provides a practical and relevant assessment of the added value of microbial inoculants following composting.

**Table 1:** Composting and seedling growth experimental treatments with different microbial inoculants.

Treatment	Soil	SMS soil amendment	
	Ratio (%)	Ratio (%)	Microbial inoculant
CK	100	-	-
C1	90	10	-
C2	90	10	*Bacillus subtilis*
C3	90	10	*Azotobacter chroococcum*
C4	90	10	*Paenibacillus mucilaginosus*
C5	90	10	Mixed microbial inoculant (V(*Bacillus subtilis*): V(*Azotobacter chroococcum*): V(*Paenibacillus mucilaginosus*) = 1:1:1)

*Stage 1: High-Temperature Composting*: Dry SMS was mechanically crushed and sieved (particle size *φ* < 0.5 cm), and water was subsequently added to adjust the moisture content of the compost to 60%. Urea was added to achieve an initial C/N ratio of roughly 30:1. Composting was carried out in seven identical rectangular foam boxes (80 cm × 100 cm × 100 cm; length × width × height) with a 10-cm wall thickness. No external microbial inoculants were added during this phase; instead, the process relied on indigenous microbial populations present in the SMS to drive aerobic fermentation. Once the pile temperature exceeded 50°C, manual aeration was performed to maintain oxygen availability. Turning frequency was set at twice per week during the first 2 weeks, and once per week in the third week. Stage 1 was terminated when the pile temperature approached ambient levels, after a total duration of 21 days.

*Stage 2: Ambient-Temperature Composting*: At the end of Stage 1, a microbial inoculant (1.0% v/v; concentration: 1.8 × 10^9^ CFU/mL) was uniformly applied to the compost material. The pile temperature was maintained below 40°C to prevent thermal inhibition of the introduced microbial strains. Aeration was maintained by turning the pile twice weekly. Stage 2 concluded after 21 days, when the compost temperature stabilized at ambient levels. The overall composting cycle lasted 42 days.

### 2.5 Seedling establishment and maintenance

With reference to previous research, different SMS soil amendments were mixed with degraded soil at a ratio of 1:9 and used to grow *P. sylvestris* (Zhou et al., 2021). Each treatment was conducted in triplicate. Two-year-old seedlings with healthy growth, intact root systems, and no signs of pests or diseases were selected and transplanted into nutrient pots, each with a volume of 2.65 L (15 cm × 15 cm × 15 cm). No chemical fertilizers, herbicides, or pesticides were applied throughout the experiment. At the end of the growth period, seedling morphological and physiological traits were recorded to evaluate treatment effects.

### 2.6 Measured responses

#### 2.6.1 Soil sample collection

Surface (0–20 cm) soil samples were collected using the five-point method to ensure representativeness across the study area after plant growth. Before analysis any visible impurities or debris in the soil were meticulously removed to avoid impurity of the results. Sample plates then were cleaned to prevent contamination, and the cleaned samples placed into sterile, self-sealing bags for safe transportation and handling. To guarantee the integrity of these samples, they were temporarily stored in a refrigerator at 4°C until further processing. After returning the samples to the laboratory, the researcher divided each one into two separate parts for conducting different kinds of test. For the first, the physicochemical properties of soil including texture, structure and nutrient content were assessed. The freely dried under controlled conditions, and then finely ground, this portion of the soil. A consistent particle size (crucial for accurate analysis) was ensured by sieving the soil through a 100 mesh screen. After this preparation, a soil sample was kept 4°C to avoid changes on the soil sample, before performing further laboratory procedures. The second lot of soil was left for microbial analysis. This helped us to better understand both the composition of the microbial community and the health of the biological soil. Both samples were then stored at −80°C to preserve microbial integrity, as microbial activity during storage could otherwise degrade or alter the microbial community structure. Under these conditions, these samples were maintained until such time when these were needed for further examination so as to accurately study the biological properties of the soil.

#### 2.6.2 Analysis of physicochemical properties

To assess soil physical properties, a container of known mass (W1) was filled with a fixed substrate volume (W2), saturated with water (W3), then drained for 10 h to obtain the final weight (W4) (Blake and Hartge, 1986). The following formulas were used:


$$\text{bulk density}=\frac{w2-w1}{w1}$$

$$\text{total porosity}=\frac{\left(w3-w1)-(w2-w1\right)}{v}\times 100\%$$

$$\text{water}-\text{holding porosity}=\frac{\left(w4-w2\right)}{v}\times 100\%$$

The soil pH and electrical conductivity (EC) were measured using the method following ASTM guidelines (ASTM International, 2019). The extraction solution was prepared by adding 50 mL of DI to 10 g of sample, mixing well, and then shaking (175 rpm, 40 min). A pH meter (PB-10, Sartorius, Germany) and EC meter (DDS-11A, Leici, China) were used to measure the pH and EC, respectively.

The total nitrogen (TN) content was determined using a Kjeldahl nitrogen analyzer (SPAD 502, KONIC MINOLA, Japan) (ISO, 1995; Ouyang et al., 2013), the total potassium (TK) content was obtained using a flame spectrophotometer (ISO, 1976; Asp et al., 2022) (6400A, Rainbow, China), and the total phosphorus (TP) content was calculated following the ammonium molybdate colorimetric method (ISO, 1983). The alkaline nitrogen (AN) content was determined by hydrolyzing the H_3_BO_3_ adsorbed NH_3_ in the soil with 2 g of NaOH (Hussain and Malik, 1985). The available potassium (AK) was extracted using 1 mol·L^−1^ ammonium acetate (CH₃COONH₄, 1:10 w:v) and quantified by atomic absorption spectroscopy (Li et al., 2018; NY/T 889-2004, 2004). The Olsen method was used to determine the available phosphorus (AP) in the soil (Olsen et al., 1954).

#### 2.6.3 Quantitative determination of microorganisms

The validity of microbial inoculation after Composting period was confirmed by selective medium viable bacteria counting method. The specific methods were as follows: composting samples were taken, diluted with 0.85% NaCl saline, centrifuged after shaking to disperse the microorganisms, and the supernatant was taken for gradient dilution and inoculated on the corresponding culture media (Hedges, 2002). Among them, *B. subtilis*, *A. chroococcum* and *P. mucilaginosus* used cycloheximide-added Tryptic Soy Agar (TSA), Ashby’s Medium and NBRIP medium, respectively (De Oliveira et al., 2011; Zhang et al., 2020). The specific incubation temperature and time for each Strain is shown in Supplementary Table S4.

The CFUs in the soil were determined following the dilution plate counting method (ASTM International, 2020; Wang W. et al., 2020). For each treatment, 10 g of soil was taken and diluted with sterile water to different multiples (bacteria, fungi, and actinomycetes were diluted to 10^3^, 10^2^, and 10^1^, respectively) and then inoculated into the corresponding culture medium. Colonies were enumerated after 3–5 days of incubation at 30°C, and the microbial concentration was quantified as CFU g^−1^.

#### 2.6.4 High-throughput sequencing analysis

Soil DNA was extracted using a kit (DcP336, Tiangen) and assessed for concentration, purity, and integrity via gel electrophoresis and a NanoDrop spectrophotometer. 16S rRNA and ITS sequences were sequenced using Miseq libraries with specific primers (338F/806R, ITS1F/ITS2R) (Gao et al., 2021). All extraction and sequencing procedures were completed on the China Scientific Compass testing platform.

#### 2.6.5 High-throughput sequencing analysis

Raw sequences were quality-filtered and assembled using the QIIME2 program with the DATA2 and Vsearch tools (Rognes et al., 2016; Bolyen et al., 2019). Sequences were clustered into operational taxonomic units (OTUs) at a 97% similarity threshold as described in a previous study (Gao et al., 2021). In addition, the UPARSE method was used to enhance the sensitivity and speed of chimera detection (Eisenhauer et al., 2011; Edgar, 2013). All data analyses were performed using QIIME2 and R language packages, including “ggplot2” (Wickham, 2016). The *α*-diversity indices were calculated, including the Chao1, Shannon, and Simpson indices (Schloss et al., 2009). Non-metric multidimensional scaling (NMDS) analysis was performed to analyze the *β*-diversity of soil microorganisms under different treatments at the OTU level based on the Bray–Curtis distance (Looft et al., 2012). The species distribution histogram revealed the top 10 and 20 species of bacteria and fungi in terms of relative abundance at the phylum and genus levels, and the other species were treated as a group.

#### 2.6.6 Seedling growth measurement

After the yearly growth cycle had ceased, the production parameters of each plant were measured, including the plant height, ground-level stem diameter (GLSD), number of leaves (NL), and chlorophyll content (Chl). The plant height above ground level was measured using a measuring tape, while the GLSD was determined at 5 cm above ground level using Vernier calipers (Wang et al., 2021). The Chl content of the leaves was obtained using a chlorophyll meter (KONIC MINOLTA, Japan), and the average of five different parts of the stem (top, upper middle, middle, lower middle, and bottom) was selected for measurement (Fei et al., 2006).

### 2.7 Statistical analyses

Microsoft Excel 2016 (Microsoft Corporation) was used to organize and analyze soil physicochemical properties, nutrient contents, and plant physiological indicators (Microsoft Corporation) was utilized. This software facilitated the organization and basic analysis of the data. SPSS 19.0 (IBM) was then employed to statistically analyze the significant differences *p* < 0.05 between the replicate samples and to conduct Pearson’s correlation analysis, which helped identify relationships among the measured variables. To explore the intricate connections between the soil microbial community and the physicochemical characteristics, Canoco 5.0 (Microcomputer Power) software was used for multivariate analysis, providing a deeper understanding of how these factors interact. For a more advanced exploration of the relationships between soil properties, microbial diversity, and plant physiological indicators, SEM was conducted using the lavaan package in the R programming language (Rosseel, 2012). This approach allowed for the construction of a comprehensive model that highlighted the complex interdependencies among these variables. Lastly, Origin 2018 (OriginLab) was employed to create high-quality visual representations, such as bar charts, box plots, and heat maps, which facilitated the interpretation and presentation of the data.

## 3 Results and discussion

### 3.1 Impact of various SMS soil amendments on soil properties

**Figure 1:**
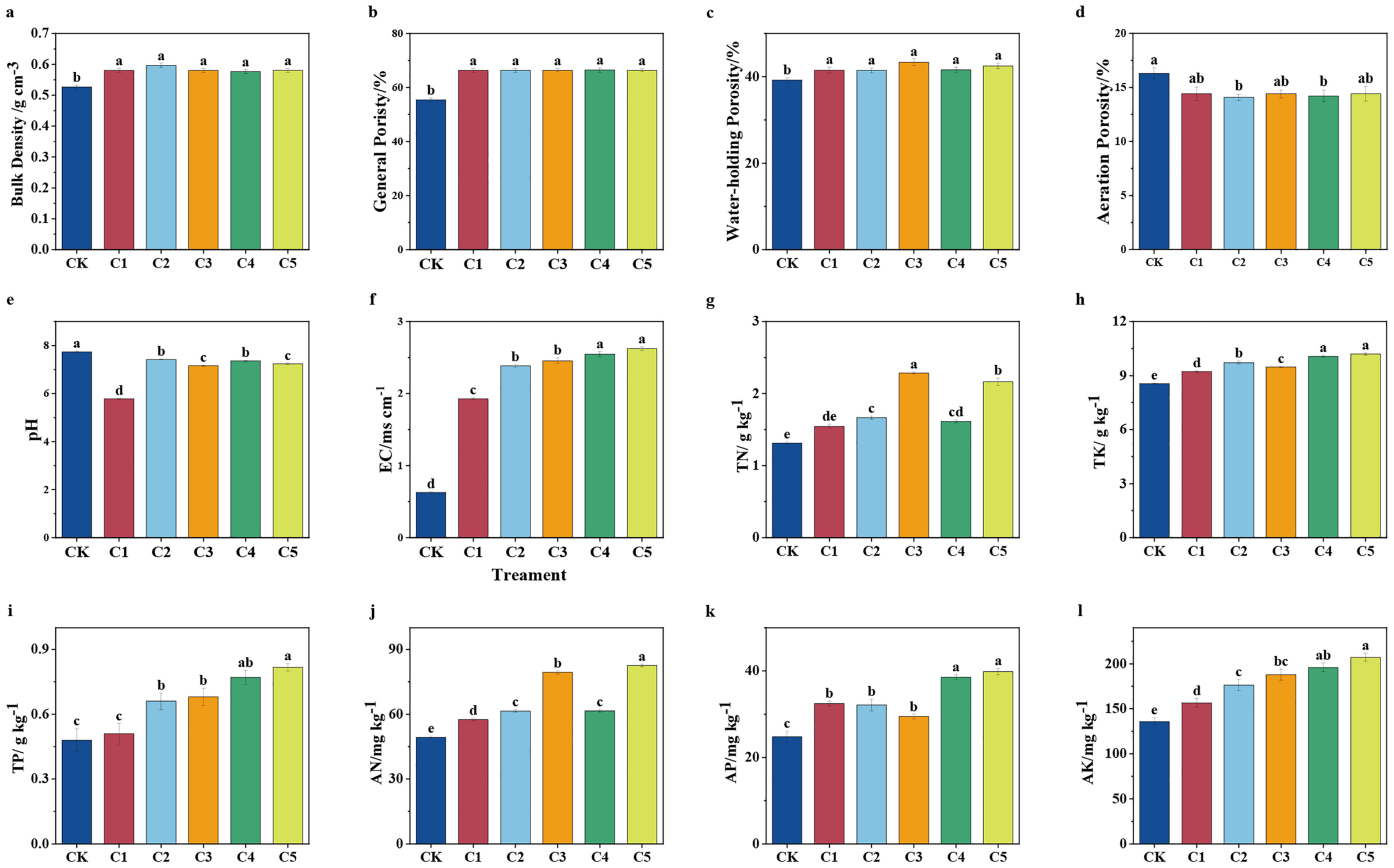
Effect of composting on the physicochemical properties of spent mushroom substrate (SMS). SMS bulk density **(a)**, general porosity **(b)**, water-holding porosity **(c)**, aeration porosity **(d)**, pH **(e)**, electrical conductivity (EC) **(f)**, total nitrogen (TN) **(g)**, total potassium (TP) **(h)**, total phosphorus (TP) **(i)**, available nitrogen (AN) **(j)**, available phosphorus (AP) **(k)**, and total potassium (TK) **(l)**. Different letters denote statistically significant variations among treatments at *p* < 0.05 level.

Figures 1a–g illustrate how different SMS-based soil amendments (CK, C1–C5) affect soil physical and chemical properties, including bulk density, porosity (total, water-holding, and aeration), pH, and electrical conductivity. The compost maturity stage and initial properties of SMS significantly influenced the results. As compost matures, microbial activity decomposes organic matter, affecting soil properties. Figure 1a shows the increase in bulk density after SMS treatment. Figures 1b,c illustrate increased total and water-holding porosity, respectively. Figure 1d reveals a shift in pH toward neutrality, and Figure 1e shows a slight decrease in aeration porosity. These changes may result from moisture loss and nutrient breakdown during composting, which improved soil porosity and water–air balance, ultimately affecting soil EC (Zhang S. et al., 2023). *Bacillus subtilis* secretes enzymes and antibiotics, suppresses pathogens, regulates pH, promotes beneficial microbes, and improves microbial community structure (Li et al., 2022). *Azotobacter chroococcum* fixes nitrogen and produces exopolysaccharides, improving soil structure and supporting microbial activity (Chen et al., 2023). *Paenibacillus mucilaginosus* solubilizes phosphorus and iron, increasing their bioavailability and shifting competitive dynamics among microbes to favor beneficial communities (Dasila et al., 2023). Collectively, these microbial inoculants improve soil biology and positively influence microbial community structure. This was aligned with the findings of Meng (2019), who found that composted digestate mixed with SMS improved the physical properties of degraded soil, which in turn increased crop yields. This effect may also be attributed to ammonium production and phenolic compound degradation during composting, which reduced soil pH, alleviated alkalinity, and influenced microbial community structure (Nie et al., 2024).

Figure 1h presents increased TP, Figure 1i shows elevated TK, Figure 1j demonstrates higher AN, and Figures 1k,l indicate enhanced AP and AK, respectively, particularly under C5 treatment. The C5 treatment consistently demonstrated the highest levels of TP, TK, AN, AP, and AK, suggesting enhanced nutrient availability and a more effective amendment profile. In comparison, C4 also improved nutrient levels, particularly for TP, AP, TK, and AK, though to a lesser extent than C5. These differences may be attributed to the differential fixation of elements by the different types of microbial inoculants added to SMS. Nitrogen-fixing bacteria enhanced humification and increased microbial enzyme activities, promoting the mineralization of organic matter and increasing the availability of soil nitrogen. Potassium-solubilizing bacteria facilitate the breakdown of potassium-bearing minerals, thereby releasing potassium ions into the soil. These findings highlight the practical applications of using SMS as a soil amendment in enhancing nutrient content. Specifically, the C5 treatment, with its superior microbial inoculants, significantly enhanced soil nutrient levels, contributing to improved soil remediation efficiency and enhanced crop yields. Previous studies have shown that microbial inoculants like *B. subtilis* can facilitate nutrient mobilization and improve soil health (Li H. et al., 2023).

After considering the improved soil structure and nutrient content, we explored the potential factors. We also acknowledge the significant role of the compost maturity stage and SMS’s initial properties in shaping the observed outcomes. As compost matures, microbial activity drives the decomposition of organic matter, affecting the soil’s physical and chemical properties (Sun et al., 2021; Huang et al., 2022). In addition to compost maturity, the initial properties of SMS—such as organic matter content and microbial load—also significantly influence its effectiveness as a soil amendment. These factors influence the microbial community composition and overall soil health, further contributing to improved soil fertility and plant growth (Meng, 2019). Thus, our results underscore the importance of both compost maturity and the initial characteristics of SMS in influencing soil improvement processes.

Overall, MMI SMS soil amendment significantly increased soil’s TN, TP, TK, AN, AP, and AK. This increase in soil nutrients aligns with Lou et al.’s findings, which showed similar improvements after applying SMS to farmland. This effect might be attributed to the activation of thermophilic microorganisms in the mixed compost, which subsequently mineralized the organic matter in SMS and transformed it into humus (Li et al., 2019). Additionally, the introduction of MMI significantly boosted the nutrient levels in the degraded soils, demonstrating a clear improvement in soil fertility (Lou et al., 2017). This was aligned with He (2022), who found that an MMI containing *A. chroococcum*, *B. subtilis*, *Micromonospora*, and *Streptomyces alboflavus* can enhance carbon conversion and nitrogen fixation when used to convert food waste into fertilizer. The application of SMS enhanced soil properties, including nutrient content, microbial diversity, and water retention capacity. This has significant implications for large-scale agricultural practices, as the use of SMS as a soil amendment could reduce reliance on chemical fertilizers, promote soil health, and improve crop yields in degraded soils. These findings support the potential for SMS to be used as a sustainable amendment for improving soil fertility and health. Future research should focus on optimizing the application of mixed microbial inoculants in SMS composting to maximize their benefits for sustainable agriculture.

### 3.2 Effects of different SMS soil amendments on soil microbial communities and diversity

#### 3.2.1 Cultivable microbial biomass

In this study, the cultivable microorganisms in each treatment were dominated by bacteria, followed by *actinomycetes* and fungi. Following the incorporating SMS, the number of CFUs was notably greater than that of the CK treatment. Specifically, substantial variations in the count of viable bacteria were observed among treatments C1 to C5. As shown in Table 2, the cultivable microbial biomass (CFU g^−1^) varied significantly among treatments. The C5 treatment demonstrated the highest bacterial and fungal abundances, with counts of 9.15 × 10^6^ and 4.84 × 10^5^ CFU g^−1^, respectively, while the *actinomycete* population peaked in the C3 treatment at 3.76 × 10^6^ CFU g^−1^. This may be due to SMS altering soil’s physicochemical properties, thereby affecting microbial growth and metabolism (Liu et al., 2019). Furthermore, the increased microbial diversity in the C5 treatment, driven by the microbial inoculants, promotes soil health and nutrient cycling. Additionally, the introduction of beneficial microbes and an enhanced carbon source promoted the growth of native soil microorganisms, likely influenced by SMS’s initial properties (Paula et al., 2017).

**Table 2:** Impact of various spent mushroom substrate (SMS) treatments on soil microorganisms.

Treatment	Microbial category		
	Bacteria (×10^6^ cfu g^−1^)	Fungi (×10^5^ cfu g^−1^)	Actinomycetes (×10^5^ cfu g^−1^)
CK	2.95 ± 0.16e	1.38 ± 0.19e	11.36 ± 0.48e
C1	6.26 ± 0.18d	2.59 ± 0.23d	25.48 ± 0.35e
C2	5.85 ± 0.32d	3.25 ± 0.26c	26.35 ± 0.51e
C3	6.86 ± 0.27c	3.15 ± 0.32c	37.57 ± 0.46e
C4	7.25 ± 0.14b	3.85 ± 0.29b	25.43 ± 0.55e
C5	9.15 ± 0.12a	4.54 ± 0.35a	26.38 ± 0.49e

Variations in lowercase letters denote statistically significant differences among the treatments (*p* < 0.05).

#### 3.2.2 Diversity and richness of the microbial community

**Figure 2:**
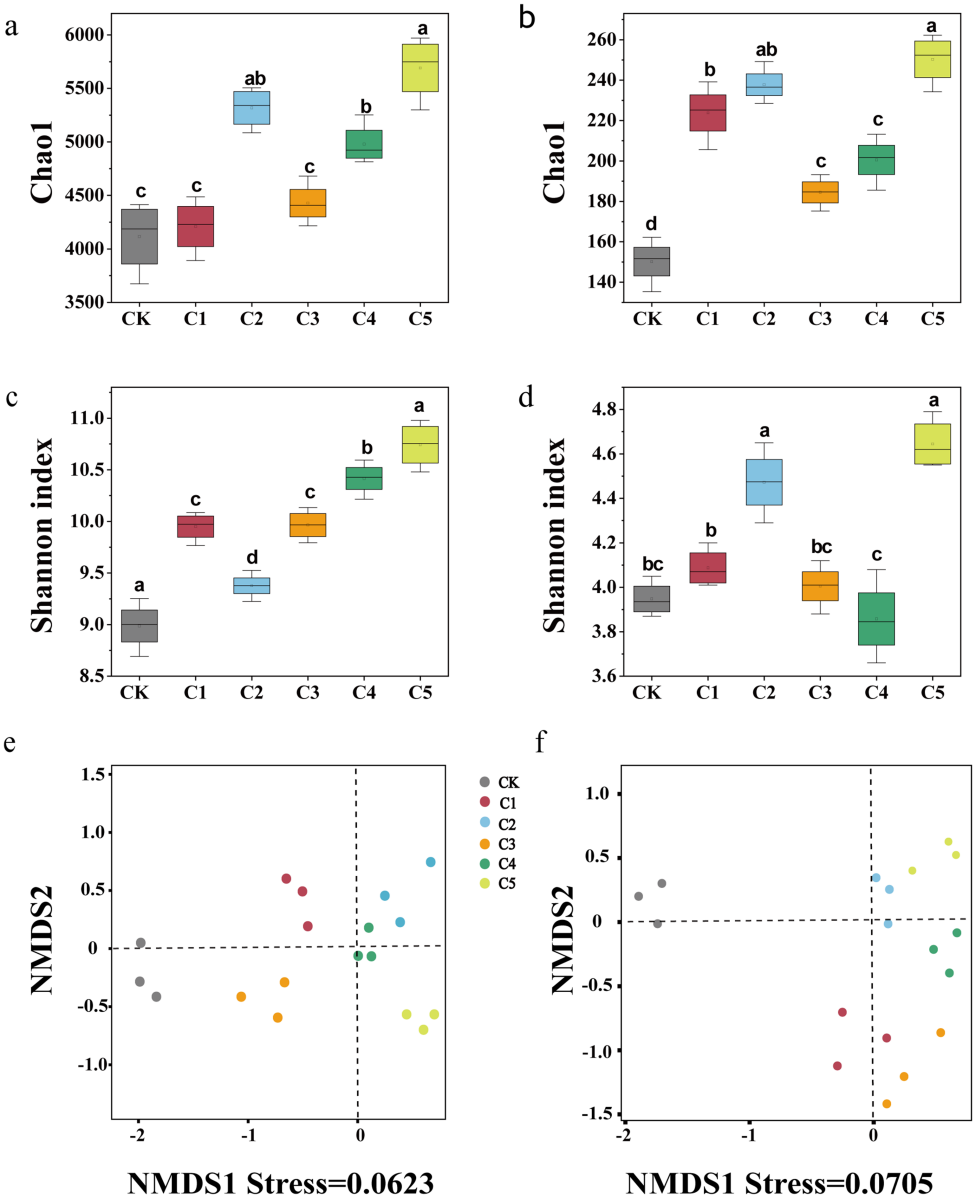
The impact of spent mushroom substrate (SMS) on α and β diversity of bacterial **(a,c)** and fungal **(b,d)** communities in the soil was assessed. Each value represents the average of three biological replicates (±SE). NMDS analysis was conducted on bacterial **(a)** and fungal **(b)** communities at the OTU level,utilizing Bray–Curtis distances. Points stand for samples, and various colors indicate various treatments. The distance between points reflects the extent of variance. NMDS analysis is considered reliable when the stress is less than 0.2.

As shown in Figure 2, the alpha diversity of bacterial and fungal communities—measured using the Chao1 richness index and the Shannon diversity index at 97% OTU similarity level—varied significantly across SMS treatments. Notably, the C5 treatment exhibited the highest microbial diversity, reflecting its strong effect on enhancing soil microbial richness and evenness. In terms of the bacterial *α* diversity, microbial inoculants significantly affected the diversity indices among treatments. The richness (Chao1) of treatment C1 was not significantly different from that of CK, while the Chao1 and Shannon indices of treatments C2–C5 increased by 2.56–26.81% and 16.58–36.34%, respectively (Figures 2a,b). In terms of the fungal α diversity, the diversity metrics of treatments C1–C5 were more than those of the control treatment (CK), with the diversity index of treatment C5 being the highest. Compared with CK, the Chao1 and Shannon indices increased by approximately 149 and 2.28, respectively (Figures 2c,d). NMDS analysis showed significant shifts in soil microbial communities, with CK’s bacterial and fungal communities distinct from others. This suggests that the microbial inoculants significantly influenced the community dynamics, causing a clear divergence between the control and the treated soil samples, with the treatments forming separate clusters. The spatial separation of samples in the NMDS plots reflects the differences in microbial diversity and community composition resulting from the application of the different treatments.

**Figure 3:**
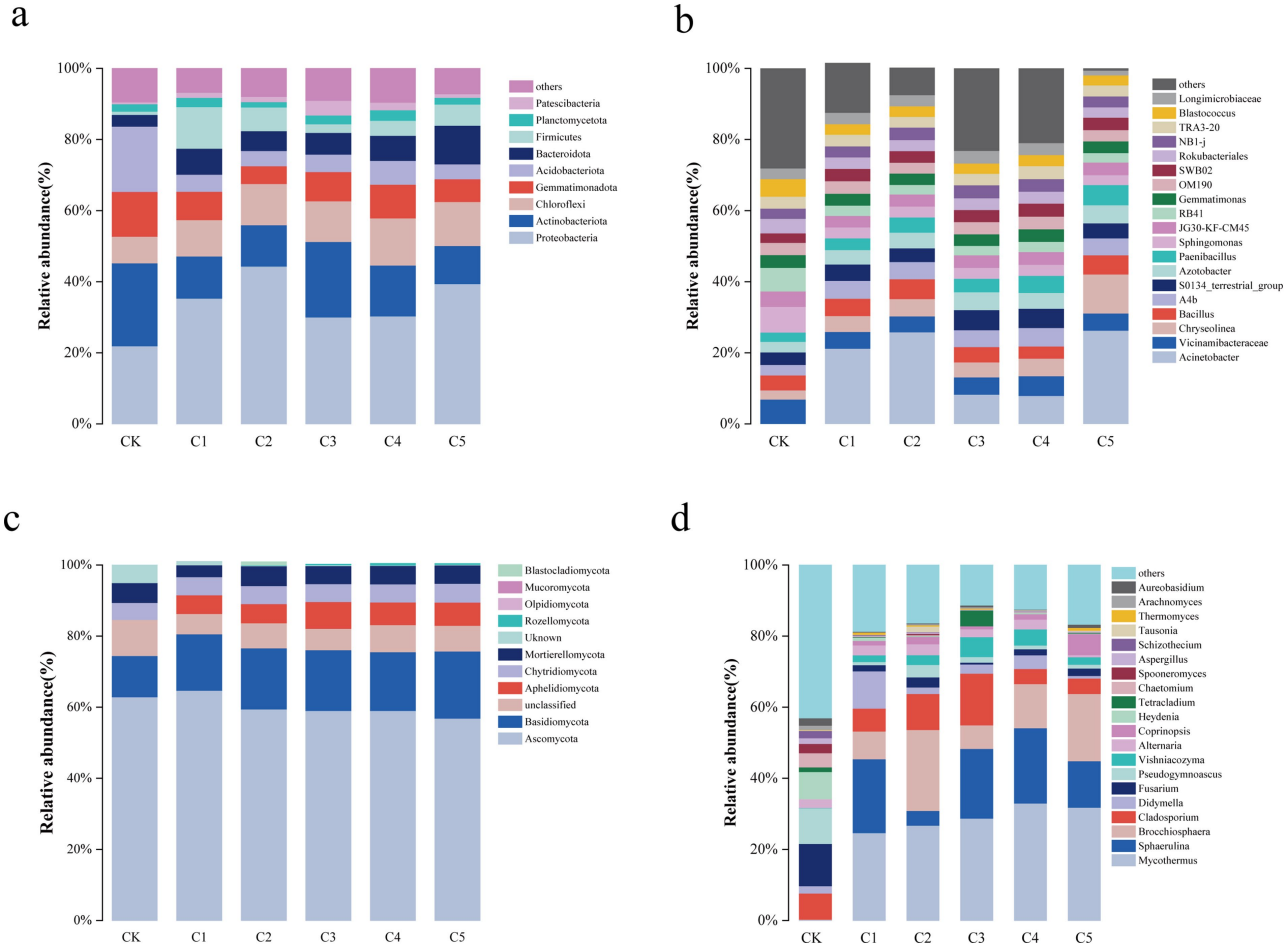
Soil microorganism abundances by phylum **(a,b)** and genus **(c,d)** across various SMS treatments.

Several studies have reported the positive effects of microbial inoculants, particularly MMI, in soil amendments. These inoculants have been shown to improve soil structure, enhance nutrient cycling, and increase microbial diversity (Zhou et al., 2020; Li et al., 2022). However, our study extends these findings by critically comparing the effectiveness of mixed microbial inoculants in different composting stages and soil types, addressing gaps in previous research regarding their long-term application and stability in agricultural settings. In our study, we observed a significant increase in microbial diversity, particularly in treatment C5, which is consistent with the findings of previous studies, but provides additional insights into the effects of microbial inoculants at different stages of composting. Researchers have demonstrated that the long-term use of synthetic fertilizers exclusively reduces microbial diversity in soils, whereas the application of organic fertilizers promotes diversity (Romaniuk et al., 2011). The increased diversity observed in the C2–C5 treatments, particularly the C5 treatment, can be attributed to the synergistic effects of microbial inoculants in promoting microbial proliferation and nutrient cycling. The higher Chao1 and Shannon indices in these treatments suggest enhanced soil microbial diversity, which is beneficial for soil health and plant growth (Shi et al., 2019). This increase in microbial diversity is positively correlated with improved nutrient availability and plant growth, particularly through enhanced soil–microbe–plant interactions (Adeniji et al., 2024). The microbial functional parameters (Chao1 and Shannon index (H′)) increased with the addition of SMS, with the most significant increase noted in the C5 treatment (Figure 3). In addition, the diversity of bacteria changed more than that of fungi, possibly because the bacterial community was more sensitive to soil amendments, a finding that was in line with Yang’s study (Yang et al., 2024). The elevated microbial abundance may be attributed to the pH regulation, which altered the microbial living environment. The nutrient-rich C5 treatment likely improved microbial carbon use, supporting the growth of r-strategy microbes (Wu et al., 2023). Adding multiple microorganisms introduced extra carbon and diverse nutrients, promoting microbial growth, reproduction, and metabolism (Zhang X. et al., 2023). The NMDS results revealed that the soil microbial communities of different SMS treatments were more distinctly differentiated (Figure 3). Taken together, the C5 treatment exhibited the highest diversity index, which indicated that the MMI SMS soil amendment significantly enriched the microbial community of degraded soil and enhanced the ability of the soil to resist harmful pathogens.

#### 3.2.3 Alterations in the structure of the microbial community

As illustrated in Figure 3, SMS amendments significantly altered the composition of soil microbial communities at both the phylum and genus levels. Notably, the C5 treatment led to an increased relative abundance of *Proteobacteria* among bacteria and *Basidiomycota* among fungi, indicating a favorable shift in microbial community structure. No significant changes at phylum/genus levels, but C5 treatment reduced *Actinobacteria/Acidobacteria* and increased *Proteobacteria/Bacteroidota*. Specifically, Figure 3a illustrates bacterial phylum-level composition with increased Proteobacteria in C5. Figure 3c displays elevated *Bacillus* and *Azotobacter* abundance at the genus level. *Proteobacteria*, as a dominant bacterial phylum, play a pivotal role in soil restoration due to their high metabolic versatility, rapid growth, and involvement in key biogeochemical cycles. Many members of this phylum, such as *Azotobacter*, *Pseudomonas*, and *Bacillus*, contribute to nitrogen fixation, phosphate solubilization, and organic matter decomposition. These functions enhance nutrient availability in degraded soils, promote plant growth, and improve soil structure. Additionally, *Proteobacteria* secrete phytohormones (e.g., IAA) and antimicrobial compounds, which help plants resist stress and suppress soil-borne pathogens (Wang et al., 2024; Zhou et al., 2025).

As shown in Figure 3b, the six most abundant fungal phyla identified at the phylum level were *Ascomycota*, *Basidiomycota*, *Aphelidiomycota*, *Chytridiomycota*, and *Mortierellomycota*. The *Ascomycota* phylum’s abundance remained unchanged, while the relative abundances of phyla *Basidiomycota* and *Chytridiomycota* were highest in the C5 treatment. The inclusion of SMS notably altered the composition and relative abundance of genera, and the relative abundances differed among the treatments. Among fungal genera, *Mycothermus*, *Sphaerulina*, *Brocchiosphaera*, *Cladosporium*, *Didymella*, and *Fusarium* became the dominant genera (Figure 3d). Similarly, *Basidiomycota* fungi, especially ectomycorrhizal and saprotrophic groups like *Mycothermus* and *Coprinopsis*, contribute significantly to soil aggregation and organic matter turnover. They produce extracellular enzymes such as lignin peroxidases and cellulases that break down complex plant residues, promoting humus formation and improving soil texture and water-holding capacity. Their mycelial networks also enhance soil porosity and serve as pathways for nutrient and water transport to plants. Furthermore, *Basidiomycota* interact symbiotically with plant roots, improving nutrient uptake, particularly phosphorus and micronutrients, and increasing plant tolerance to environmental stressors (Manici et al., 2024).

In addition, this study found that a notable alteration in the soil microbial community structure with MMI SMS soil amendment treatment (Figure 3).

The rapid nutrient release and improved soil moisture likely resulted from the MMI SMS amendment, increasing *Proteobacteria* and *Bacteroidota* while decreasing oligotrophic groups like *Actinobacteria* and *Acidobacteria* (Bhardwaj et al., 2024). In nutrient poor conditions, these latter groups were less abundant and the C5 treatment resulted in a surge in abundance of *Bacillus* spp. within the *Proteobacteria* phylum. Interestingly, this increase is very helpful to improve the disease resistance in degraded soils (Wang et al., 2022). Importantly, the increase of *Azotobacter* spp. in MMI SMS soil amendment may also enhance the process of nitrogen accumulation and transformation in degraded soils.

In this study, the relative abundance of *Ascomycota* changed little, which may have occurred because the physicochemical properties of all treatments were supportive of the proliferation of saprophytic fungi. *Mortierellomycota* is a fast-growing saprophyte that accounts for 20% of soil fungi and plays a positive role in controlling diseases in degraded soil. The relatively high abundances of *Basidiomycota* and *Chytridiomycota* in the C5 treatment may be explained by the fact that *Basidiomycota* is an ectomycorrhizal fungus that can accelerate the decomposition of cellulose in SMS, as well as the circulation of substances. This aligns with Huang et al.’s findings on fungal community-driven humification (Huang et al., 2022). At the genus level, higher levels of *Vishniacozyma* and *Coprinopsis* align with Meng et al.’s findings that *Basidiomycota* efficiently utilize nutrients (Meng et al., 2019).

*Proteobacteria*, as a dominant bacterial phylum, play a pivotal role in soil restoration due to their high metabolic versatility, rapid growth, and involvement in key biogeochemical cycles. Many members of this phylum, such as *Azotobacter*, *Pseudomonas*, and *Bacillus*, contribute to nitrogen fixation, phosphate solubilization, and organic matter decomposition. These functions enhance nutrient availability in degraded soils, promote plant growth, and improve soil structure. Additionally, *Proteobacteria* secrete phytohormones (e.g., IAA) and antimicrobial compounds, which help plants resist stress and suppress soil-borne pathogens (Li et al., 2022; Zhang S. et al., 2023).

### 3.3 Effects of different SMS soil amendments on plant growth

**Figure 4:**
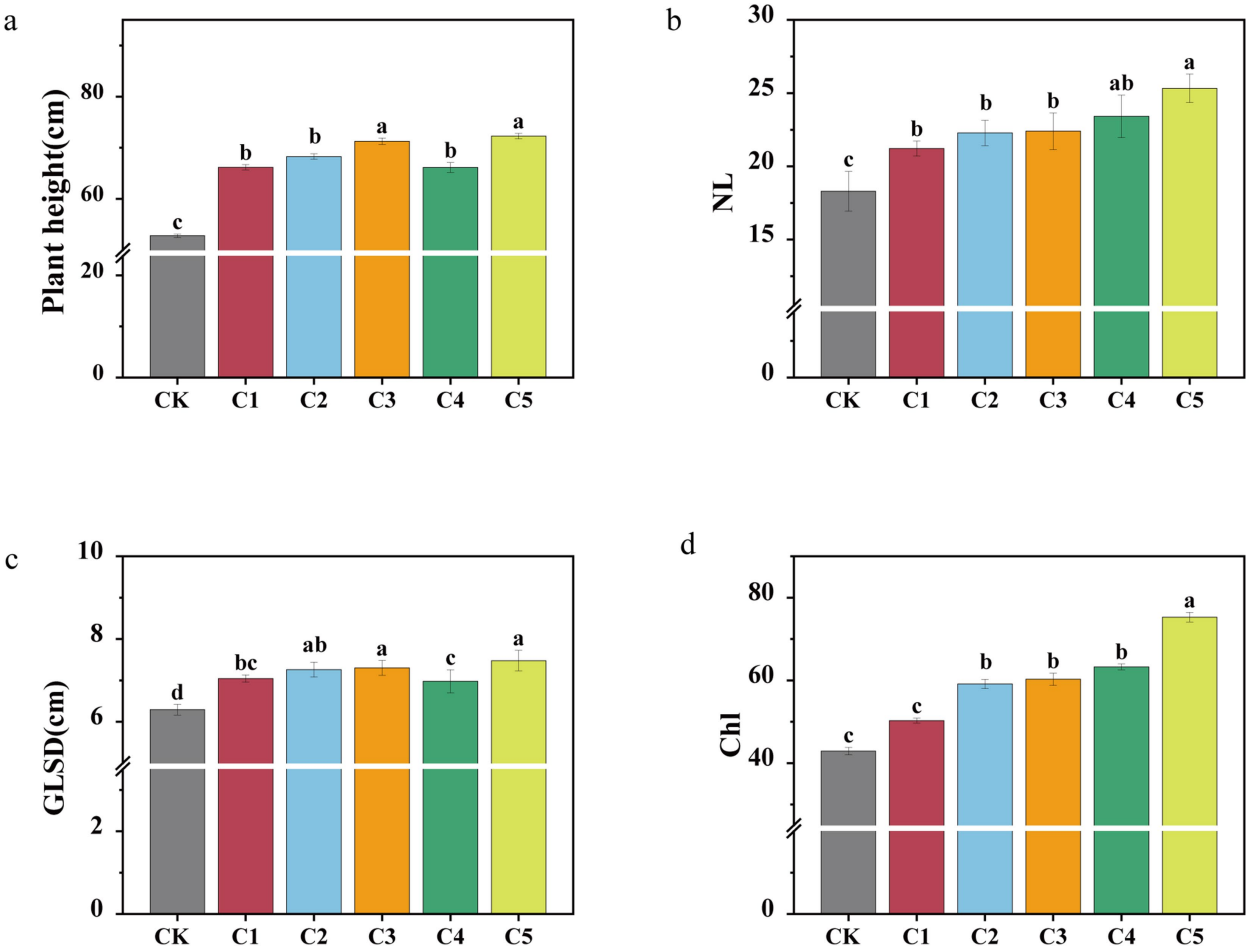
Effect of different spent mushroom substrate (SMS) treatments on plant physiological indicators: plant height **(a)**, ground-level stem diameter (GLSD) **(b)**, number of leaves (NL) **(c)**, and chlorophyll (Chl) content of leaves **(d)**. Distinct letters denote notable disparities among treatments at the *p* < 0.05 level.

Applying SMS as a soil amendment could significantly promote plant growth, but plant physiological indicators varied with different treatments. Figures 4a–d illustrates the effects of different SMS treatments on key plant growth parameters, including plant height, ground-level stem diameter (GLSD), number of leaves (NL), and chlorophyll content (Chl). The C3 and C5 treatments showed the greatest plant height and stem diameter (Figures 4a,b), while NL was notably higher in C5 and C4 compared to other groups (Figure 4c). The highest Chl content was also observed in C5 (Figure 4d). Overall, C5 outperformed other treatments and had the most significant positive impact on seedling growth.

The results showed that SMS improved plant physiological indicators compared to CK, though the effects varied across treatments (Figure 4). This difference may have been because the addition of SMS improved the rhizosphere environment of degraded soil. This supports previous conclusions that the application of SMS in tomato cultivation can boost crop yields (Khalil et al., 2024). More importantly, C5 showed the best plant physiological performance, likely due to its superior nutrient profile and microbial composition, which most effectively enhanced the rhizosphere environment (He, 2022). Previous studies have shown that microbial inoculants significantly enhance composting processes by accelerating the decomposition of organic matter and improving compost quality (Meng, 2019; He, 2022). In particular, studies have highlighted the importance of microbial diversity and the interactions between different microbial strains in driving compost maturity and enhancing soil health (Zhou et al., 2020). Consistent with these findings, our results indicate that microbial inoculants, particularly mixed strains, significantly improved the quality of the compost, which in turn contributed to enhanced plant growth and overall health. However, although pot experiments provide valuable data in a controlled environment, their limitations lie in the inability to fully replicate the complexity of field conditions (Gheysari et al., 2021). The spatial constraints of pots may affect root growth, which could influence plant growth patterns and soil health responses. Additionally, pot experiments cannot simulate real-world factors such as weather fluctuations, pests, and other external variables that are significant in field settings. These limitations underscore the need for future field validation of the findings.

Despite these positive findings, several limitations should be acknowledged. This study was conducted under pot experiment conditions, which offer controlled environments but may not fully simulate the complexity of natural field conditions. Factors like weather fluctuations, soil variability, pests, and long-term ecological interactions are hard to simulate in pot experiments. The short experiment duration limits assessment of SMS’s long-term effects on soil and microbes. The absence of field trials also limits the generalizability of the results. Future research should include long-term, multi-location field experiments to evaluate the effectiveness and stability of SMS amendments under practical agricultural conditions. Additionally, this study did not evaluate the post-application survival or physiological activity of the microbial inoculants in the soil environment. Although this limitation may influence the interpretation of microbial community dynamics to some extent, it does not compromise the observed improvements in soil fertility, microbial diversity, and plant growth. Future research should incorporate microbial tracking methods, such as qPCR or metatranscriptomic analysis, to validate the persistence and functional roles of inoculants *in situ*.

### 3.4 Relationships between soil properties, the microbial community, and plant physiological indicators

#### 3.4.1 Relationships between soil properties and microbial communities

**Figure 5:**
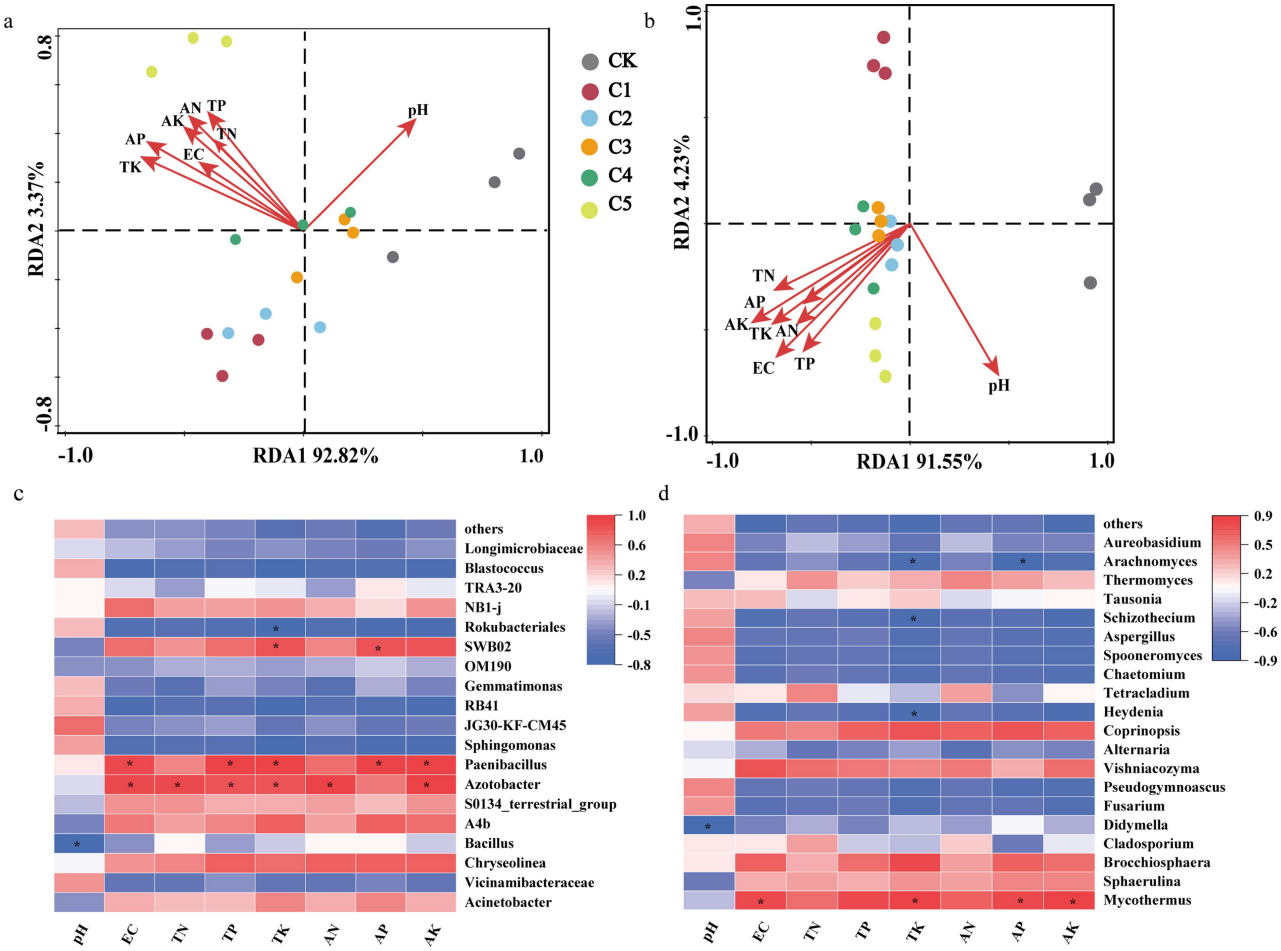
Relationships between soil properties and microbial community composition across various spent mushroom substrate (SMS) treatments. **(a)** Redundancy analysis (RDA) of the relationships between soil properties and bacterial communities at the operational taxonomic unit (OTU) level. **(b)** RDA of the relationship between soil properties and fungal communities at the OTU level. **(c)** Pearson correlation analysis of soil bacterial communities and soil variables. *p < 0.05; **p < 0.01. **(d)** Pearson correlation analysis of fungal communities and soil variables. *p < 0.05; **p < 0.01.

To investigate the factors that most significantly influence soil microbial communities, redundancy analysis (RDA) was performed, as illustrated in Figure 5. The results show that soil properties such as EC, TN, TP, and pH strongly influenced both bacterial and fungal community composition across treatments. This analysis examined the relationships between environmental variables and the 10 most abundant microbial genera. In the bacterial community, the first two RDA axes together explained 96.19% of the total variance, with RDA axis 1 accounting for 92.82% and axis 2 for 3.37%. The pH and the concentration of key soil elements emerged as the dominant environmental factors shaping bacterial variation (Figure 5a). For the fungal community, RDA axis 1 and axis 2 explained 91.55 and 4.23% of the total variation, respectively. The levels of EC, TN, TP, TK, AN, AP, and AK exhibited positive correlations with the fungal community in treatments C2–C5. Conversely, soil pH had a negative influence on both bacterial and fungal populations (Figure 5b). RDA showed that different physicochemical factors had varying effects on microbial structure. Treatment C5 had a notable impact on the soil environment, mainly due to changes in soil nutrient content. Microbial growth is strongly influenced by physicochemical factors that affect microbial physiological and biochemical processes. These changes, in turn, have a significant impact on the diversity of microbial communities (Feng et al., 2018). It is therefore postulated that the process of SMS composting modifies the physicochemical properties of degraded soil, leading to subsequent shifts in the structure and diversity of the soil’s microbial community.

The Pearson correlation analysis indicated that the genus *Azotobacter* spp. under *Proteobacteria* was strongly positively correlated with EC, TN, TP, TK, AN, and AK (*p* < 0.05), while *Paenibacillus* spp. showed significant positive correlations with EC, TP, TK, AP, and AK (*p* < 0.05) (Figure 5c). In the phylum *Proteobacteria*, *Bacillus* spp. was significantly negatively in correlation with pH. This may be because genera such as *Bacillus*, which play an important role in restoring degraded soils, thrive in near-neutral pH conditions (Zakavi et al., 2024). According to Zhang et al., *Azotobacter* spp., *Paenibacillus* spp. and *Bacillus* spp. are important beneficial rhizosphere microorganisms. These genera participate in the nitrogen and phosphorus cycles in the soil, enhance plant disease resistance (Zhang S. et al., 2023). Combined with the information shown in Figure 1, it can be seen that the application of SMS can increase the soil nutrient contents and change the community structure of soil microbial bacteria.

As shown in Figure 5d, in the phylum *Basidiomycota*, the genus *Mycothermus* displayed significant positive correlations with soil EC values and nutrients (TK, AP, and AK) (*p* < 0.05). The soil phylum *Basidiomycota*, particularly the genus *Thermomycothermus*, can produce a variety of thermostable cellulases during the high-temperature composting of SMS. These enzymes can decompose cellulose and hemicellulose in SMS. After entering the soil, the genus *Mycothermus* can participate in the mineralization of soil organic matter, carbon and nitrogen cycling, and the formation of soil aggregates (Wang X. et al., 2020).

#### 3.4.2 Relationships between soil properties, the microbial community, and plant physiological indicators

**Figure 6:**
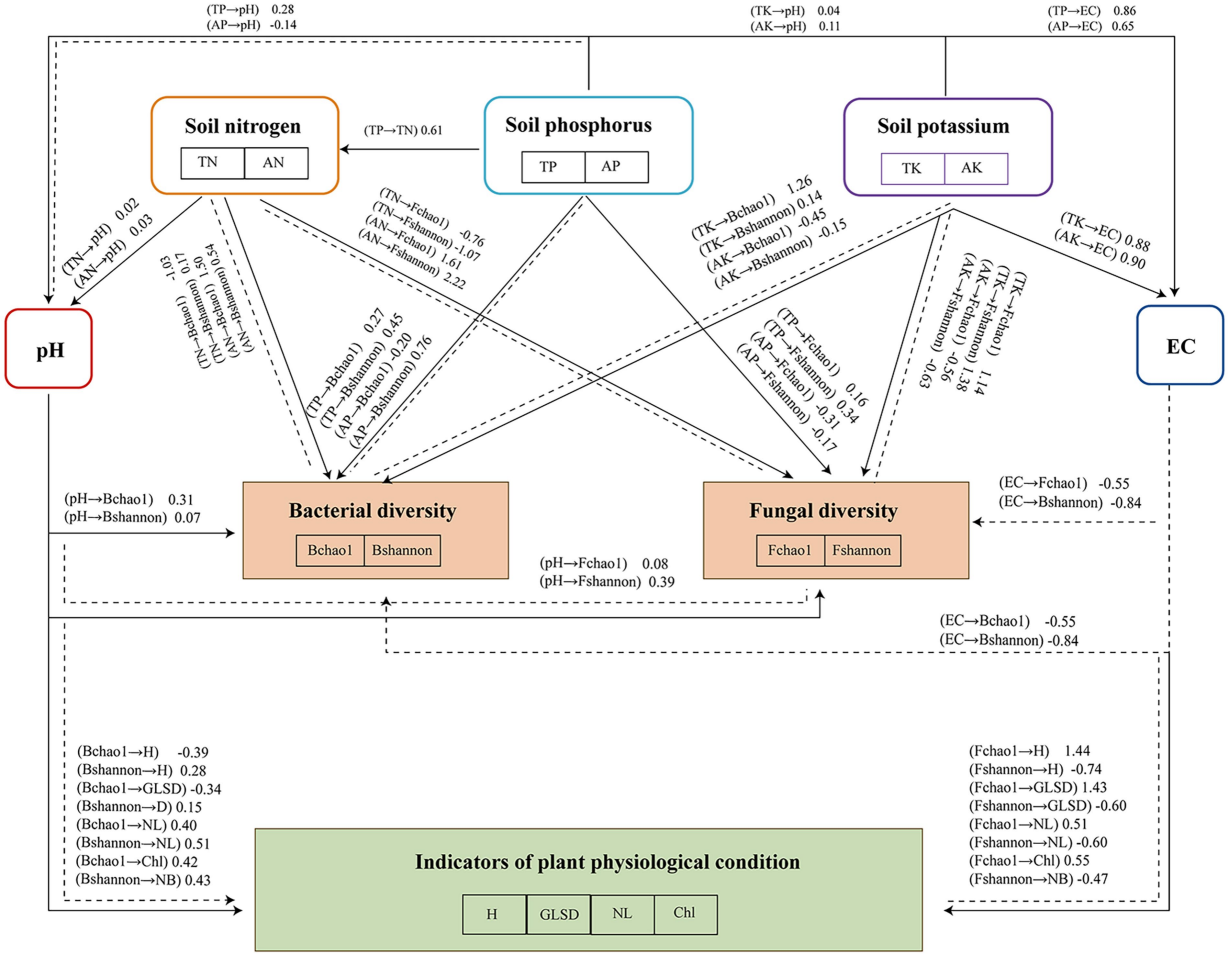
Structural equation model (SEM) of the physicochemical properties of soil, nutrient content, and plant physiological indicators. Bchao1: bacterial Chao1 index; Bshannon: bacterial Shannon index; Fchao1: fungal Chao1 index; Fshannon: fungal Shannon index; H: plant height; GLSD: ground-level stem diameter; NL: number of leaves; and Chl: chlorophyll content of leaves.

A structural equation model (SEM) was developed to explore how soil physicochemical properties influence microbial community composition and plant physiological indicators (Figure 6). The SEM illustrates the pathways through which soil properties and microbial diversity jointly influence plant growth parameters under different SMS treatments. The results illustrated that the plant physiological indicators were encouragingly associated with bacterial richness and evenness (Chao1 and Shannon indices). For fungi, indicators were positively related to richness (Chao1), but negatively to evenness. Suggesting a shift in fungal community structure as plant health improved. Soil amendments, especially SMS, improve soil structure and fertility, thereby enhancing plant growth. SMS amendments enhance microbial activity, particularly by promoting the growth of beneficial rhizosphere microorganisms that support soil health and nutrient cycling. The interaction between soil amendments, microbial activity, and plant health underscores the importance of sustainable soil management practices for improving long-term agricultural productivity (Huang, 2022). In addition, both the Chao1 and Shannon indices of bacteria and fungi were supportively associated with pH and unfavorably related with EC. High EC increases salinity, which harms plant growth. The results aligned with those of Shen et al. (2016), who found that elevated soil salinity was detrimental to the growth of the microorganisms. The SEM revealed inconsistencies in the impacts of soil nutrients on the microbial community, as confirmed by the study of Tao et al. (2022). We found that the TN had an inverse relationship with bacterial Shannon index and both the fungal Chao1 and Shannon indices, illustrating that higher TN correlates to less microbial diversity for these groups. On the other hand, NH₄^+^ had a positive correlation with both bacterial and fungal Chao1 indices, implying that NH₄^+^ might fuel the richness of microbial community. A positive correlation of microbial diversity in both bacterial and fungal populations with TP was considered as having beneficial effect of TP on soil microbial diversity. Conversely, higher AP was linked to reduced microbial richness, suggesting it may negatively affect microbial communities. Bacterial and fungal diversity were positively affected by the TK content, whereas bacterial and fungal diversity were negatively affected by the AK content. Consequently, SMS soil amendments can regulate soil microbial diversity by modulating nutrient accumulation patterns, physicochemical properties, and enhancing plant growth in degraded soils. To enhance the relevance and applicability of the study’s findings, future research should expand to field trials that validate the effects of different SMS treatments under real agricultural conditions. Large-scale experiments across diverse soil types and climatic zones are recommended to assess the impacts on soil nutrient cycling, microbial diversity, and plant growth (Li et al., 2020). Moreover, future studies should explore the integration of SMS with other soil management strategies—such as crop rotation, organic fertilization, or reduced chemical input systems—to identify optimal approaches for enhancing soil fertility (Ma et al., 2025). Long-term field trials will also be essential to evaluate the sustained effects of SMS on soil structure, nutrient availability, and overall ecosystem health. Moreover, the findings of this study have important implications for sustainable land management. The use of spent mushroom substrate (SMS) and microbial inoculants not only improves degraded soil quality but also provides a viable strategy to recycle organic waste, thus contributing to circular economy practices. By transforming agricultural by-products into valuable soil amendments, this approach reduces the reliance on synthetic fertilizers, minimizes environmental pollution, and promotes resource-efficient farming systems. These benefits align with global efforts to promote regenerative agriculture and sustainable soil use, especially in regions facing land degradation and limited soil fertility. Future policy and practice may benefit from integrating SMS-based composting into broader land restoration frameworks.

## 4 Conclusion

This study demonstrates that composted SMS combined with mixed microbial inoculants (MMI) significantly enhances soil properties, nutrient availability, and microbial diversity in degraded soils, which in turn promoted the growth of *Pinus sylvestris* seedlings—reflected in improved height, stem diameter, leaf number, and chlorophyll content. These benefits are attributed to the synergistic effects of *B. subtilis*, *A. chroococcum*, and *P. mucilaginosus* in promoting nutrient cycling, regulating pH, and shaping beneficial microbial communities. The two-stage composting process—thermophilic fermentation followed by targeted inoculation—was essential for compost maturity and soil amendment effectiveness. While these findings are promising, they are based on pot experiments; thus, long-term field trials are necessary for validation. To further advance this strategy, future research should incorporate microbial functional profiling (e.g., PICRUSt, FUNGuild) and network analyses to uncover keystone taxa and functional interactions. Multi-omics approaches such as metatranscriptomics or metabolomics can deepen our understanding of microbial dynamics under SMS treatments, thereby guiding the development of more effective and sustainable soil remediation strategies.

## Data Availability

The original contributions presented in the study are publicly available. This data can be found here: https://www.ncbi.nlm.nih.gov/sra, accession number: PRJNA1308473.
