# Supplementary material for: Synergistic effects of beneficial microbial inoculants and SMS-amendments on improving soil properties and Pinus seedling growth in degraded soils
Source: Front Microbiol. 2025 Sep 1;16:1608689. doi: 10.3389/fmicb.2025.1608689 (PMC12434066; doi:10.3389/fmicb.2025.1608689)
Supplement: Supplementary file 1 [file Data_Sheet_1.pdf]

Supplementary Material

Table S1. Before the experiment, the physicochemical properties and nutrient content of the soil were measured from 0 to 20 cm.

Soil basic properties	
pH	7.98
EC	0.63
Total nitrogen (TN, g kg^{-1})	1.31
Total phosphorus (TP, g kg^{-1})	0.48
Total potassium (TK, g kg^{-1})	8.56
Alkaline nitrogen (AN, mg kg^{-1})	49.22
Available phosphorus（AP, mg kg^{-1}）	24.78
Available potassium (AK, mg kg^{-1})	135.78

Table S2. Before the experiment, the physicochemical properties and nutrient content of the soil were measured from 0 to 20 cm.

microbial inoculant	Culture Medium Formula	
B. subtilis	peptone	10.0 g/L
	yeast extract	5.0 g/L
	NaCl	10.0 g/L
	pH	7.0–7.1
A. chroococcum and P. mucilaginosus	KH_2PO_4	0.4 g/L
	K_2HPO_4	0.1 g/L
	D-gluconic acid sodium	5.0 g/L
	MgSO4	0.2g/L
	NaCl	0.1 g/L
	CaCl2	0.02g/L
	$FeCl_3$	0.01g/L
	yeast extract powder	0.8mg/L
	$Na_2MoO_4 \cdot 2H_2O$	0.002 mg/L
	pH	6.8–7.0

Table S3. Physicochemical properties and nutrient content of SMS.

Soil basic properties	
Moisture (%)	15.2
pH	4.32
EC	0.56
Total nitrogen (TN, g kg-1)	2.58
Total phosphorus (TP, g kg-1)	1.21
Total potassium (TK, g kg−1)	12.26
Alkaline nitrogen (AN, mg kg-1)	101.26
Available phosphorus (AP, mg kg-1）	52.58
Available potassium (AK, mg kg-1)	218.32

Table S4. Culture Conditions and Medium Formulations for Microbial Strains Used in Viable Plate Count Analysis

Microbial Strain	Selective Medium	Medium Composition (per L)	Supplement	Incubation Temperature (°C)	Incubation Time (h)
B. subtilis	Tryptic Soy Agar (TSA)	Tryptone 15.0 g, Soy peptone 5.0 g, NaCl 5.0 g, Agar 15.0 g	Cycloheximide 0.1 g (added after sterilization)	30	24–48
A. chroococcum	Ashby's Nitrogen-Free Medium	Mannitol 10.0 g, K_2HPO_4 0.2 g, $MgSO_4 \cdot 7H_2O$ 0.2 g, NaCl 0.2 g, $CaSO_4$ 0.1 g, $FeCl_3$ trace, Agar 15.0 g	None	28	48–72
P. mucilaginosus	NBRIP Medium	Glucose 10.0 g, $Ca_3(PO_4)_2$ 5.0 g, $MgSO_4 \cdot 7H_2O$ 0.25 g, $MgCl_2 \cdot 6H_2O$ 5.0 g, KCl 0.2 g, $(NH_4)_2SO_4$ 0.1 g, Agar 15.0 g	None	30	48
